# Multicenter Comparison of Nucleic Acid Amplification Tests for the Diagnosis of Rectal and Oropharyngeal Chlamydia trachomatis and Neisseria gonorrhoeae Infections

**DOI:** 10.1128/JCM.01363-21

**Published:** 2022-01-19

**Authors:** Barbara Van Der Pol, Max Chernesky, Charlotte A. Gaydos, Edward W. Hook, Ajith M. Joseph, Katherine Christensen, Rodney Arcenas, Alexander Boutwell, Harold C. Wiesenfeld, Stephanie N. Taylor, Kenneth H. Mayer, Matthew R. Golden, Jeanne Moncada, Dan Jang, Julius Schachter, the ExGen Study Group, Kristal Aaron

**Affiliations:** University of Alabama at Birmingham; University of Alabama at Birmingham; Becton, Dickinson and Company, BD Life Sciences – Integrated Diagnostic Solutions; Abbott, Medical Affairs; Roche Diagnostic Solutions; Roche Diagnostic Solutions; Abbott, Medical Affairs; Becton, Dickinson and Company, BD Life Sciences – Integrated Diagnostic Solutions; Becton, Dickinson and Company, BD Life Sciences – Integrated Diagnostic Solutions; University of Alabama at Birmingham; a University of Alabama School of Medicine, Birmingham, Alabama, USA; b McMaster University, St Joseph’s Healthcare, Hamilton, Ontario, Canada; c Johns Hopkins School of Medicine, Baltimore, Maryland, USA; d Abbott, Medical Affairs, Lake Forest, Illinois, USA; e Becton, Dickinson and Company, BD Life Sciences – Integrated Diagnostic Sciences Solutions, Sparks, Maryland, USA; f Roche Diagnostic Systems, Pleasanton, California, USA; g University of Pittsburgh School of Medicine and Magee-Womens Research Institute, Pittsburgh, Pennsylvania, USA; h Louisiana State University Health Sciences Center, New Orleans, Louisiana, USA; i Fenway Health, Boston Massachusetts, USA; j University of Washington, Seattle, Washington, USA; k University of California-San Francisco, San Francisco, California, USA; Marquette University

**Keywords:** *Chlamydia trachomatis*, *Neisseria gonorrhoeae*, rectal infection, oropharyngeal infection, molecular diagnostics, extragenital, extragenital STI, molecular methods, sexually transmitted infections

## Abstract

Research using nucleic acid amplification tests (NAATs) have repeatedly found rectal and oropharyngeal infections with Chlamydia trachomatis and Neisseria gonorrhoeae to be common and potentially more difficult to treat than genital infections. Unfortunately, public health and patient care efforts have been hampered by the lack of FDA-cleared NAATs with claims for anorectal or oropharyngeal samples. At the time of the initiation of this study, no commercially available assays had these claims. We formed a novel partnership among academic institutions and diagnostic manufacturers to address this public health need. From May 2018 through August 2019, we recruited 1108 women, 1256 men, and 26 transgender persons each of whom provided 3 anal and 3 oropharyngeal swab specimens. The 3 anal swabs were pooled into a single transport tube as were the 3 oropharyngeal swabs. The performance of each of three study assays was estimated by comparison to the composite result and relative to one another. Percent positivity for chlamydia was 5.9 and 1.2% from anal and oropharyngeal specimens, respectively, compared to 4.2 and 4.1% for gonorrhea. Sensitivity for chlamydia detection ranged from 81.0 to 95.1% and 82.8 to 100% for anal and oropharyngeal specimens, respectively. Gonorrhea sensitivity ranged from 85.9 to 99.0% and 74.0 to 100% for anal and oropharyngeal samples, respectively. Specificity estimates were ≥ 98.9% for all assays, organisms, and sample types. Although there was heterogeneity between sensitivity estimates, these assays offer better ability to detect extragenital infections than culture and potential solutions for providing appropriate sexual health care for populations in which these infections are of concern.

## INTRODUCTION

For decades, it has been appreciated that Chlamydia trachomatis (CT) and Neisseria gonorrhoeae (NG) could infect the oropharynx and rectum (1). In the field of sexually transmitted infections (STI), these are commonly referred to as extragenital infections, despite the fact that ocular and other infections are also non-genital in nature, and we will use that nomenclature throughout this report. Interest in extragenital infections increased when Kent and colleagues reported that nucleic acid amplification testing (NAAT) of oropharyngeal or anorectal swab specimens showed that these infections were far more common than previously thought (2). The number of infections identified among men who have sex with men (MSM) from whom samples were collected from all mucosal sites were often as high as double the number identified using the standard practice of testing only for urethral infection (2, 3). In 2009, the Centers for Disease Control and Prevention (CDC) recommended annual CT and NG screening of rectal and oropharyngeal sites among MSM. This recommendation was included in the 2010 Sexually Transmitted Disease Treatment Guidelines (4) and during the same meetings, the optimal method for detecting these infections was determined to be NAAT, which was described in the recommendations for laboratory diagnosis of chlamydia and gonorrhea (5). Both of these recommendations were made despite the fact that no assays were cleared by the Food and Drug Administration (FDA) for use with anorectal or oropharyngeal specimens at that time. Further research has shown that oropharyngeal CT and NG infections are more common than previously believed in all persons practicing oral sex and that rectal infections are also common among sexually active women even in the absence of reported receptive rectal intercourse (6–8). Clinical studies also suggest that some extragenital infections (rectal chlamydia, oropharyngeal gonorrhea) may be more difficult to treat than genital infections (9, 10). Further studies of the relevance and relative importance of potential routes of exposure are topics of ongoing investigation. However, integration of extragenital CT and NG testing into routine medical care have been hampered by the lack of FDA-cleared assays for detection of oropharyngeal and anorectal STI (11) and the limited number of laboratories with the resources to perform rigorous validation studies in order to provide this service to their clients.

This study represents a unique collaboration between scientists from three diagnostic manufacturers and eight academic institutions in an effort to respond to that need. The clinical trial was designed to generate data that could be used in support of seeking FDA review and clearance for the use of oropharyngeal and anorectal swab specimens to diagnose CT and NG infections with commercially available NAAT assays.

## MATERIALS AND METHODS

### Study development and management.

This study design was developed initially by a small group of academic collaborators. Once a consensus draft was developed, representatives of assay manufacturers were asked to review the protocol and make suggestions related to their regulatory needs. Then, a joint group of academicians and sponsors met with the FDA to provide the agency with our proposed study design. The agency provided feedback, including the use of a universal transport system for pooled specimens and appropriateness of sensitivity and specificity estimates based on the study design. The agency also outlined the studies that would need to be performed internally by each manufacturer to demonstrate equivalence of their collection device to the sampling scheme used in this study. In order to provide fair and unbiased oversight, the University of Alabama (UAB) STD Clinical Research Organization (CRO) managed data capture and cleaning, oversight of data collection and protocol deviations, data storage, and all analyses. Sponsors were allowed to attend site visits and provide observations to the UAB STD CRO staff, but never had access to editable data.

### Eligibility.

**FIG 1:**
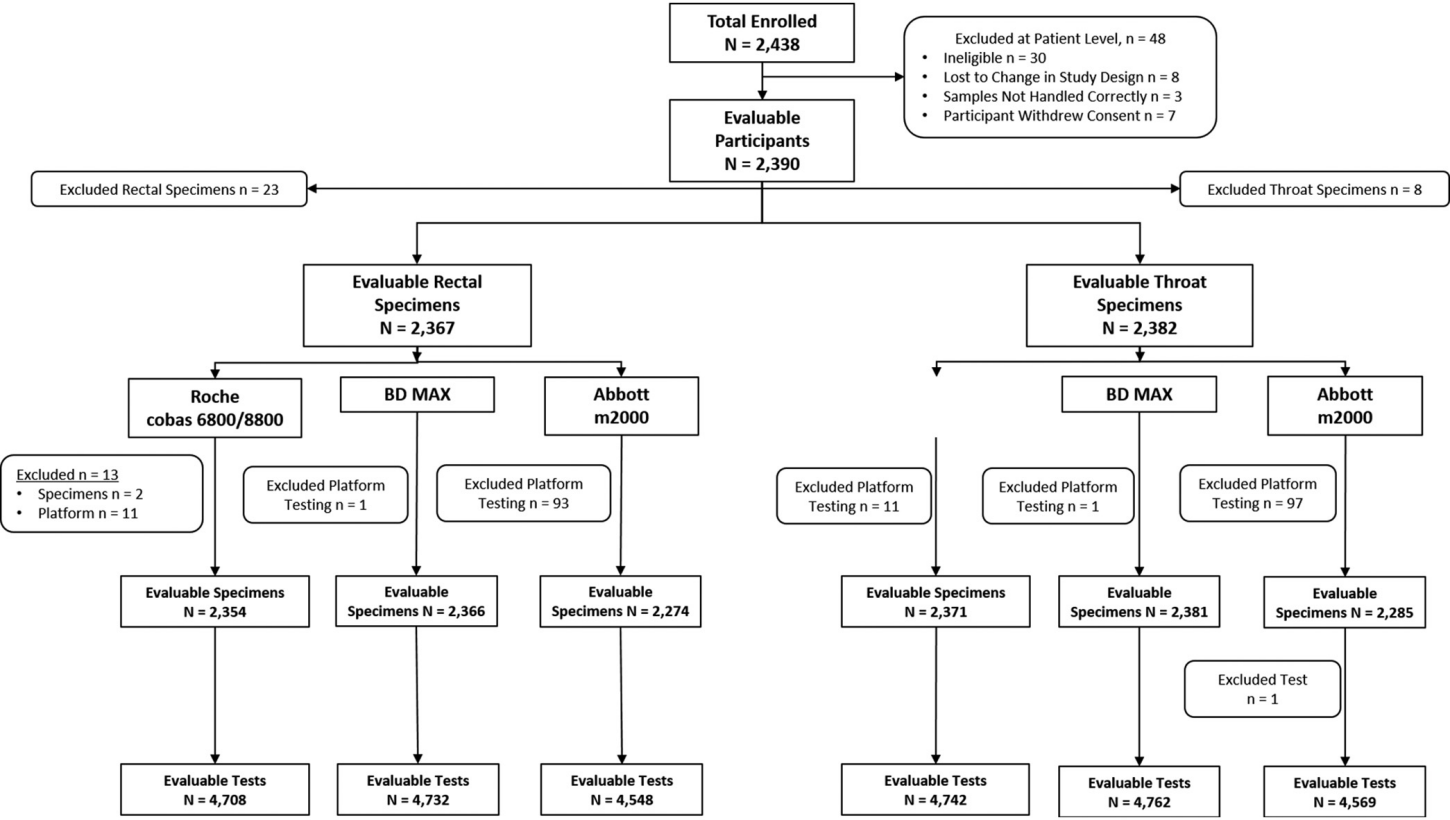
Participant disposition. Exclusions occurred at the patient level (no test results were eligible/evaluable for these participants), specimen level (no test results were eligible/evaluable for these samples), or platform level (no test results were eligible/evaluable for these specimens on a single assay). Roche refers to the cobas, BD to the MAX, and Abbott to the m2000 assays and platforms.

We recruited men and women reporting extragenital exposures, regardless of genital, rectal, or oral symptoms, attending eight outpatient clinics, where testing for sexually transmitted infections (STI) is routine. These included outpatient clinics for family planning and obstetrics/gynecology, and adolescent and sexual health clinics, such as public health clinics, STD/STI clinics, or clinics focused on sexual and gender minority health care (Table 1). Participants were ≥ 14 years old (or the youngest age of self-consent determined by local institutional policy); reported oral, anal, or vaginal sex within the previous 30 days; were willing to disclose and discuss sexual exposures; were able to speak English or Spanish; were willing and able to provide written consent; and agreed to clinician-collected oral and anorectal specimens. Exclusion criteria included having taken antibiotics active against CT or NG infection in the past 30 days; use of topical rectal product(s) in the last 24 h; or previous study enrollment. Sexual and medical histories were taken prior to sample collection to verify eligibility. Once enrolled, exclusions could occur at the participant level (all data for that participant was excluded); the sample level (no results from any platform were available for a specific sample); or the test level (where results from only a single platform were excluded because results for a specific sample were not available from that platform) due to protocol deviation/s (Fig. 1). The entire protocol was approved by the University of California, San Francisco institutional review board (IRB), and each collection site obtained local IRB study approval.

**TABLE 1:** Study sites, number recruited (n), and number positive for CT/NG DNA

Site location	Clinic type	Gender	n	CT rectal +	NG rectal +	CT oropharyngeal +	NG oropharyngeal +
Boston, MA (collection site)	HIV	Female	29	0	0	0	0
		Male	136	6 (4.4%)	3 (2.2%)	2 (1.5%)	4 (2.9%)
		Transgender	5	0	0	0	0
		Total	170	6 (3.5%)	3 (1.8%)	2 (1.2%)	4 (2.4%)
Baltimore, MD (aliquoting/testing site)	STD	Female	39	3 (7.7%)	1 (2.6%)	1 (2.6%)	1 (2.6%)
		Male	52	0	0	0	3 (1.9%)
		Transgender	0	0	0	0	0
		Total	91	3 (3.3%)	1 (1.1%)	1 (1.1%)	4 (4.4%)
New Orleans, LA (collection site)	STD	Female	56	11 (20.0%)	1 (1.8%)	1 (1.8%)	3 (5.4%)
		Male	319	31 (9.7%)	40 (12.5%)	2 (0.6%)	32 (10%)
		Transgender	13	1 (7.7%)	2 (15.4%)	0	1 (7.7%)
		Total	388	43 (11.1%)	43 (11.1%)	3 (0.8%)	36 (9.3%)
Hamilton, ONT (aliquoting/testing site)	Special adolescent STD	Female	70	3 (4.3%)	2 (2.9%)	1 (1.4%)	0
		Male	100	1 (1.0%)	1 (1.0%)	0	0
		Transgender	1	0	0	0	0
		Total	171	4 (2.3%)	3 (1.8%)	1 (0.6%)	0
Seattle, WA (collection site)	STD	Female	30	2 (6.7%)	2 (6.7%)	1 (3.3%)	2 (6.7%)
		Male	227	13 (5.7%)	14 (6.2%)	0	15 (6.6%)
		Transgender	4	0	1 (25.0%)	0	1 (25.0%)
		Total	261	15 (5.7%)	17 (6.5%)	1 (0.4%)	18 (6.9%)
San Francisco, CA (aliquoting/testing site)	Adolescent options center STD	Female	174	6 (3.4%)	1 (0.6%)	2 (1.1%)	0
		Male	267	26 (9.7%)	12 (4.5%)	8 (3.0%)	12 (4.5%)
		Transgender	1	0	0	0	0
		Total	442	32 (7.2%)	13 (2.9%)	10 (2.3%)	12 (2.7%)
Pittsburg, PA (collection site)	OB/GYN	Female	381	15 (3.9%)	5 (1.3%)	3 (0.8%)	6 (1.6%)
		Male	18	1 (5.6%)	3 (16.7%)	3 (16.7%)	3 (16.7%)
		Transgender	1	0	0	0	0
		Total	400	16 (4.0%)	8 (2.0%)	6 (1.5%)	9 (2.2%)
Birmingham, AL (aliquoting/testing site]	STD	Female	329	19 (5.8%)	8 (2.4%)	4 (1.2%)	9 (2.7%)
		Male	137	1 (0.7%)	4 (2.9%)	0	4 (2.9%)
		Transgender	1	0	0	0	0
		Total	467	20 (4.3%)	12 (2.6%)	4 (0.9%)	13 (2.8%)
All sites		Female	1,108	59 (5.3%)	20 (1.8%)	13 (1.2%)	21 (1.9%)
		Male	1,256	79 (6.3%)	77 (6.1%)	15 (1.2%)	73 (5.8%)
		Transgender	26	1 (3.8%)	3 (11.5%)	0	2 (4.0%)
		Total	2,390	139 (5.9%)	100 (4.2%)	28 (1.2%)	96 (4.1%)

### Specimen collection.

One of the difficulties of comparative analyses is collection of multiple swabs per site. While this is not generally a major barrier for vaginal swabs, when considering anorectal swabs, it becomes a more significant issue. For oropharyngeal sample collection, which if done correctly sometimes induces a gag response in patients, it is a substantial barrier. Based on the clinical experience of the team, we targeted collection of three swabs per site as feasible for most patients. Use of three swabs rather than a single sample ensured that we would have sufficient concentration of the organisms (if present) in the 10 ml of pooled sample to mimic a single swab collected in an assay transport device. Thus, each anatomic site (oropharyngeal and anorectal) was sampled using three Dacron swabs from the Abbott multi-collect specimen collection kit (Abbott Molecular, Des Plaines, IL). These swabs were validated for use with each of the assays prior to study initiation. Immediately following specimen collection, each of the three swabs was broken off at a score mark and placed in a single vial containing 10 ml of PreservCyt (Hologic, Foxborough, MA) medium. This strategy, which was arrived at in consultation with the FDA, was designed to avoid sample collection variation by pooling three samples into a single vial that could then be aliquoted for testing by each of the assays. PreservCyt was chosen as a universal medium because each assay had been previously demonstrated to be compatible with this medium. Swabs were neither lubricated nor pre-moistened before specimen collection. Clinicians collected all specimens in order to maximize quality of the repeated sampling. The three oropharyngeal specimens were collected by rotating each swab against each tonsillar pillar and the posterior pharynx. If pharyngeal exudate was present, it was also sampled. Clinicians sequentially collected three anorectal specimens by inserting the swabs 1–2.5 cm into the anal canal and rotating at least one time. Specimens discolored by stool or blood were not excluded. Thus, for each participant, a total of six extragenital samples were collected: three anal swabs into a single PreservCyt vial and three pharyngeal swabs into another vial to provide a uniform single oropharyngeal sample and a single anorectal sample for evaluation by each assay. The pooled samples were then aliquoted into manufacturers’ collection devices (Fig. S1).

### Specimen handling and testing.

Four collection-only trial sites did not process or test samples. These sites shipped specimens in PreservCyt, weekly, to one of four pre-assigned laboratories responsible for specimen aliquoting and testing (Table 1). Samples were aliquoted into each of four manufacturer’s transport tubes, and NAATs were performed using platforms available in each laboratory. Specimens were shipped to other testing labs for analysis using platforms that were not supported at their laboratory. No single laboratory performed all four NAATs used in this study. Thus, aliquoted samples were shipped weekly to a second laboratory performing NAATs not covered by the first laboratory.

For aliquoting, specimens in PreservCyt were vortexed for 10 s, and 0.5 ml was transferred to each of the transport tubes used for the following assays (Fig. S1): *m*2000 RealTi*m*e CT/NG assay (m2000; Abbott Molecular, Des Plaines, IL) (12) and the MAX CT/GC/TV (MAX; BD Integrated Diagnostic Solutions, Sparks, MD) (13). The Roche cobas 6800/8800 CT/NG (cobas; Roche Diagnostics, Indianapolis, IN) assay (14) uses PreservCyt directly and therefore, a 4 ml sterile cryovial received 0.75 ml of the PreservCyt sample for shipment to a cobas testing site. At laboratories performing testing using the cobas assay, 0.5 ml of the sample was transferred to 1.0 ml PreservCyt in a transport tube used on the cobas 6800 instrument. In order to have three results to compare to each assay, an 0.5 ml aliquot was also prepared for a fourth assay (Aptima Combo 2 CT/GC assay (AC2), Hologic, San Diego, CA (15)) the performance of which was not analyzed as part of this study. Assays were performed according to manufacturers’ instructions for swab specimens on the m2000 and MAX assays and for PreservCyt specimens with the cobas and AC2 assays.

### Analyses.

Each specimen was tested by four NAATs to minimize potential bias. When using only three tests that compare a single test to results from only two other assays, bias is related to the variability in performance of the two comparator tests when these two results are not concordant. Further, use of only two comparators could potentially generate skewed results if either of the comparators performed very well or very poorly (16). In these circumstances, sensitivity and specificity cannot be estimated and performance is measured in percent positive and negative agreement. In our study, the performance of each assay was calculated by comparison to a composite reference standard (CRS) where infection was defined by results from three other assays. Thus, for each assay, a specimen was considered positive if at least two of the other three comparator results were positive, and negative if at least two of the other three assay results were negative. Since at least two concordant results were required in order to classify samples as positive or negative, samples that generated 1) a single positive result, 2) a single negative result, and 3) an unresolvable invalid result, were unevaluable for the comparison. It is important to note that while 4 assay results were generated for each sample, the CRS comprised only the three results for which an estimate was not being calculated. Thus, when estimating the sensitivity of the m2000, results generated by that assay were not included in the CRS; only results from the MAX, cobas, and AC2 contributed to defining infection status. Similarly for estimates of MAX performance, only m2000, cobas, and AC2 results were considered for the CRS, and for cobas performance only m2000, MAX, and AC2 results contributed to the CRS. Therefore, the final number of evaluable, valid results available for comparison for each assay varied slightly as did the number of infections defined for each comparison. Table S1 shows how test results were distributed across all assays.

Using this comparator approach, consistent with FDA input, it is appropriate to generate sensitivity and specificity estimates for each of the assays in comparison with results provided by the other three tests. Therefore, outcome measures for each assay included estimates of sensitivity, specificity, and 95% confidence intervals using the Score method (17). Separate analyses were performed for each assay by specimen type (rectal and oropharyngeal), organism (CT/NG), age, gender, and symptom status. Fisher’s Exact Test was used to compare the performance estimates across the assays under evaluation with adjustment for multiple comparisons. An α = 0.05 assigned statistical significance. R was used to perform all statistical analyses (R Core Team, 2020). These analyses were performed only for the three assays that were part of the original study design and analytical plan.

## RESULTS

### Study population.

Between May 22, 2018 and August 22, 2019, we recruited 2,438 participants into the study with 2,390 evaluable participants who provided 4,749 evaluable specimens from which we generated a total of 28,061 evaluable results for the three assays under evaluation. Details about participant/sample disposition and exclusions are shown in Fig. 1. These data reflect only the three assays under evaluation as there were no exclusions applied to testing with the AC2 comparator assay. The 8 patients lost to changes in the study protocol, enrolled during pilot rollout, fell into three categories: 1 participant self-collected and the clinic reported that the process did not work well; 5 participants’ samples were collected while sites still had access to older collection kits that contained puritan swabs rather than the Abbott multi-collect swabs; and 2 participants’ specimens were inadvertently routed to a lab that ultimately could not perform testing on the required platforms. Overall, there were no unresolved (i.e., two indeterminate results for a specific specimen) results on the m2000; three male anorectal (both CT & GC), three male oropharyngeal (both CT & GC), and one oropharyngeal specimen from a transgender person (both CT & GC) on the MAX; and one male oropharyngeal (both CT & GC) on the cobas. This represents a non-result rate of <0.2% for all assays (data not shown). Other samples excluded from analysis were based on protocol deviations related to specimen testing. One site had a delay in testing on the m2000 system that resulted in the exclusion of 97 samples on the m2000 platform.

We attempted to achieve balanced enrollment of men and women. Ultimately, women comprised 46.4% (1,108/2,390) of the study population, men 52.5% (1,256/2,390), and transgender persons 1.1% (26/2,390) (Table 1). Men enrolled in the study were older than women and transgender persons (*P* < 0.001) (Table 2). Participants were predominately Black women (706/1,108, 63.7%) and White men (717/1,256, 57.1%). Genital symptoms were more commonly reported by women than men (30.1% vs 24.9%, *P* = 0.004), while rectal and throat symptoms were more commonly reported by men: 5.4% vs 1.1% and 6.5% vs 1.5%, respectively (both *P* values <0.001).

**TABLE 2:** Selected characteristics of study population

Descriptor	Female	Male	Transgender	p
N	1,108	1,256	26	
Age (mean (SD))	29.8 (9.4)	34.9 (11.9)	30.2 (11.0)	<0.001
Symptomatic				
Rectal symptoms	340 (30.7%)	419 (33.8%)	5 (19.2%)	0.102
Throat symptoms	12 (1.1%)	82 (6.5%)	2 (7.7%)	<0.001
Genital symptoms	17 (1.5%)	68 (5.4%)	1 (3.8%)	<0.001
Hispanic ethnicity	75 (6.8%)	184 (14.6%)	6 (23.1%)	<0.001
Race				
Black	706 (63.7%)	368 (29.3%)	10 (38.5%)	<0.001
White	286 (25.8%)	627 (49.9%)	10 (38.5%)	
Other	116 (10.5%)	261 (20.8%)	6 (23.1%)	

CT positivity (defined by at least 3 positive assay results) at the oropharynx was 1.2% (28/2,390) and was evenly distributed among men, women, and transgender persons (Table 1), which was lower than the 5.8% (139/2,390) positivity for CT using anorectal samples. Seven participants were CT positive at both the rectum and oropharynx representing 5.0 and 25.0% of total infections at each of these sites, respectively. Anorectal CT positivity was 6.3% and 5.3% among men and women, respectively (*P* = 0.3803). Oropharyngeal positivity was 1.2% among both male and female participants. Only 2 (0.2%) men had both CT and GC detected in the oropharynx while 15 (1.2%) and 8 (0.7%) men and women, respectively, had both CT and GC detected using the anorectal sample.

NG positivity was 4.2% (100/2390) and 4.0% (96/2390) at the anorectal and oropharyngeal sites, respectively. NG infections at both sites were identified in 32 participants representing 33.3% of oropharyngeal and 32.0% of rectal infections. NG positivity was significantly lower for women than men at both anatomic sites: anorectal NG was 1.8% compared to 6.1% for women and men and oropharyngeal NG 1.9% versus 5.8% (both *P* values <0.001). Anal positivity was higher for MSM than MSW: 8.1 vs 0.3% (*P* < 0.001) and 7.5 vs 1.7% (*P* < 0.001), for CT and NG, respectively. For oropharyngeal samples there was no difference in CT rates between MSM and MSW, but oropharyngeal NG rates were higher among MSM (6.5%) compared to MSW (3%) (*P* = 0.04).

### NAAT performance.

Among the multiple performance estimates generated in this study, we found a single significant difference by gender. The m2000 CT estimates of rectal sensitivity were 91.7% for females vs 73.3% in males (*P* = 0.006). Given the otherwise strong concordance, and because we found no evidence to show that test performance for infections at extragenital sites are substantively different between men and women, we do not present results by gender here (results by gender and self-reported sexual exposure will be reported elsewhere). Similarly, there was no difference in assay performance when evaluated by symptom status (Table S2). Thus, we present only summary tabulations of performance, regardless of gender or symptom status.

Specificity estimates were >98.9% for each of the three study assays for CT and NG (Tables 3 and 4) using either anorectal or oropharyngeal specimens. More heterogeneity was seen with sensitivity estimates. Anorectal specimens tested for CT had sensitivity estimates of 81.0, 94.6, and 95.1% for m2000, MAX, and cobas, respectively (Table 3). The cobas and MAX sensitivity estimates for CT from anorectal specimens were each significantly higher than the estimate for m2000 (both *P* values <0.001). When testing oropharyngeal specimens for CT, sensitivity estimates were 82.8, 100, and 100% for m2000, MAX and cobas, respectively. None of these estimates were significantly different among the three assays.

**TABLE 3:** NAAT Performance for detection of C. trachomatis

	m2000	MAX	Cobas
Rectal specimens			
Sensitivity 95% CI	81.0% (119/147) 73.9–86.5%	94.6% (139/147) 89.6–97.2%	95.1% (136/143) 90.2–97.6%
Specificity 95% CI	100% (2114/2115) 99.7–100%	99.5% (2164/2175) 99.10%– 99.72%	99.2% (2175/2192) 98.8–99.5%
			
Oropharyngeal specimens			
Sensitivity 95% CI	82.8% (24/29) 65.5%–92.40%	100% (28/28) 87.9%–100.00%	100% (28/28) 87.9–100%
Specificity 95% CI	99.9% (2241/2243) 99.7%–100%	99.8% (2299/2304) 99.5%–99.9%	99.8% (2313/2317) 99.6%–99.9%

**TABLE 4:** NAAT performance for detection of N. gonorrhoeae

	m2000	MAX	Cobas
Rectal specimens			
Sensitivity 95% CI	85.9% (91/106) 78.0–91.2%	91.5% (97/106) 84.7–95.5%	99.0% (100/101) 94.6–99.8%
Specificity 95% CI	99.9% (2156/2158) 99.7–100%	99.9% (2215/2218) 99.6–100%	99.3% (2222/2237) 98.9–99.6%
			
Oropharyngeal specimens			
Sensitivity 95% CI	74.0% (77/104) 64.9–81.5%	88.4% (91/103) 80.7–93.2%	100% (96/96) 96.2–100%
Specificity 95% CI	99.9% (2163/2165) 99.7–100%	98.9% (2210/2234) 98.4–99.3%	98.9% (2228/2253) 98.4–99.3%

For NG testing of anorectal specimens, the sensitivity estimates were 85.9, 91.5, and 99.0% for m2000, MAX, and cobas, respectively (Table 4). The cobas sensitivity was significantly higher than the estimate for m2000 (*P* = 0.002). For oropharyngeal samples, the sensitivity estimates were 74.0, 88.4, and 100% for m2000, MAX, and cobas, respectively. Sensitivity estimates for cobas were higher than the estimate for MAX (*P* = 0.002), and both cobas and MAX had higher sensitivity estimates than m2000 (*P* < 0.001 and *P* = 0.03, respectively).

## DISCUSSION

Research studies have generated substantial data since 2005 to show that NAAT testing can be used accurately for extragenital samples (18–20). These data have generated considerable clinical interest in oropharyngeal and rectal testing and highlight the need for FDA cleared assays that can be used to test extragenital samples without performing lengthy validation studies. In some populations, had testing been restricted to only genital specimens, 50% or more of CT or NG infections would not have been identified (11). Further, recent data suggest that some extragenital infections may fail treatment more often than genital infections (9, 21), that oropharyngeal gonorrhea infections may contribute importantly to development of gonococcal antimicrobial resistance, and that rectal STIs may increase risk for HIV acquisition and transmission (22). Despite these public health needs, diagnostic manufacturers found that the clinical trials needed to collect data supporting 510k submissions to obtain FDA clearance were prohibitively expensive and time-consuming. When this trial began, no NAAT had received FDA clearance to detect CT or GC in extragenital specimens, however, during the study enrollment period, the AC2 (Hologic) and Xpert CT/NG assay (Cepheid, Sunnyvale, CA) obtained clearance via an NIH supported project (23). The positive percent agreement (PPA) estimates generated by this study for AC2, which was used as a comparator in our study, were 88.7% (rectal CT), 88.2% (oropharyngeal CT), 96.5% (rectal GC), and 95.1% (oropharyngeal GC).

In our study, the cobas assay had excellent performance for detection of both CT and NG in anorectal and oropharyngeal specimens with sensitivity estimates ranging between 95.2% and 100% and specificity estimates >98.9%. The MAX assay was comparable or slightly less sensitive (88.4%–100%) and achieved essentially the same specificity for both organisms using either sample type. These performance estimates are similar to those reported for the other assays that received FDA clearance based on data from the NIH sponsored study (23).

Abbott’s m2000 did not perform as well as the other two assays in this evaluation nor in the NIH study mentioned above where the PPA estimates were 83.0% (rectal CT), 84.0% (oropharyngeal CT), 88.3% (rectal GC), and 84.8% (oropharyngeal GC) (24). In our study, the use of a three-way comparison and the small number of oropharyngeal CT-positive results may have limited our power to detect a statistically significant difference in performance for detection of oropharyngeal CT. This finding is in contrast to prior studies demonstrating similar performance for the m2000 in CT and NG diagnosis using genital specimens when compared to other platforms (12, 18, 25). It is important to note that all of the performance estimates are conservative given that we collected the equivalent of 1 swab into 3.3 ml of PreservCyt and 0.5 ml was placed into each of the assays, which is more dilute than what is routinely used for genital samples.

One point of concern about molecular testing for NG is related to cross-reactivity with closely related species, which is of particular concern for oropharyngeal sampling since many of these species are commensal organisms in that compartment. Assays have continued to address this issue for testing with genital sample types, and the list of closely related organisms included by each manufacturer in their exclusivity analyses (to verify no reactivity with these species) is extensive. Further, the use of three comparator tests to determine the specificity of each assay is a strength of the study that gives us confidence that the positive results are indeed a reflection of infection with N. gonorrhoeae. It is important to note that for any sample that generated two positive and two negative results (of which we saw only 10/2,390 (0.4%) results in this study), the assay being evaluated was always classified as incorrect (i.e., false negative or false positive) so the bias would be to underestimate performance. We feel confident that use of molecular testing alone, without culture or other, less sensitive detection methods, was an appropriate strategy for estimating performance for NG even from oropharyngeal specimens.

This novel study design and unique collaboration may provide a model for future studies of diagnostic tests where emergency use is important (COVID-19 pandemic is a current example) or where public health needs do not provide corporations with sufficient incentives to offset the substantial commitments required for clinical studies. One example might be seeking FDA clearance for home-collected samples for CT and NG. By sharing costs with competitors, the trials may become more affordable, without loss of competitive advantage. It is our hope that this trial will ultimately provide public health labs and clinicians with more options to diagnose anorectal and oropharyngeal CT/NG infection. Our experiment with this type of collaboration can be considered successful since Roche now has claims for anorectal and oropharyngeal specimens for the cobas 6800/8800 CT/NG assay. Abbott and BD are in the process of submitting data from this study in support of claims for extragenital sample types as well.

Many questions remain regarding recommendations for appropriate screening for oropharyngeal and rectal CT/NG infection, regarding the clinical significance and potential for complications arising from such infections and for optimal treatment. Further, self-collection of specimens will need to be evaluated to support testing that is acceptable to patients and can be performed in a variety of settings. We hope that data from this study will facilitate the approval process to make anorectal and oropharyngeal CT/NG testing more widely available.
