# Supplementary material for: Multicenter Comparison of Nucleic Acid Amplification Tests for the Diagnosis of Rectal and Oropharyngeal Chlamydia trachomatis and Neisseria gonorrhoeae Infections
Source: J Clin Microbiol. 2022 Jan 19;60(1):e01363-21. doi: 10.1128/JCM.01363-21 (PMC8769746; doi:10.1128/JCM.01363-21)
Supplement: Supplemental file 1 — Fig. S1 and Tables S1 and S2. Download JCM.01363-21-s0001.pdf, PDF file, 0.7 MB [file jcm.01363-21-s0001.pdf]

Supplementary Figures & Tables

Figure S1. Samples Pooling & Aliquoting

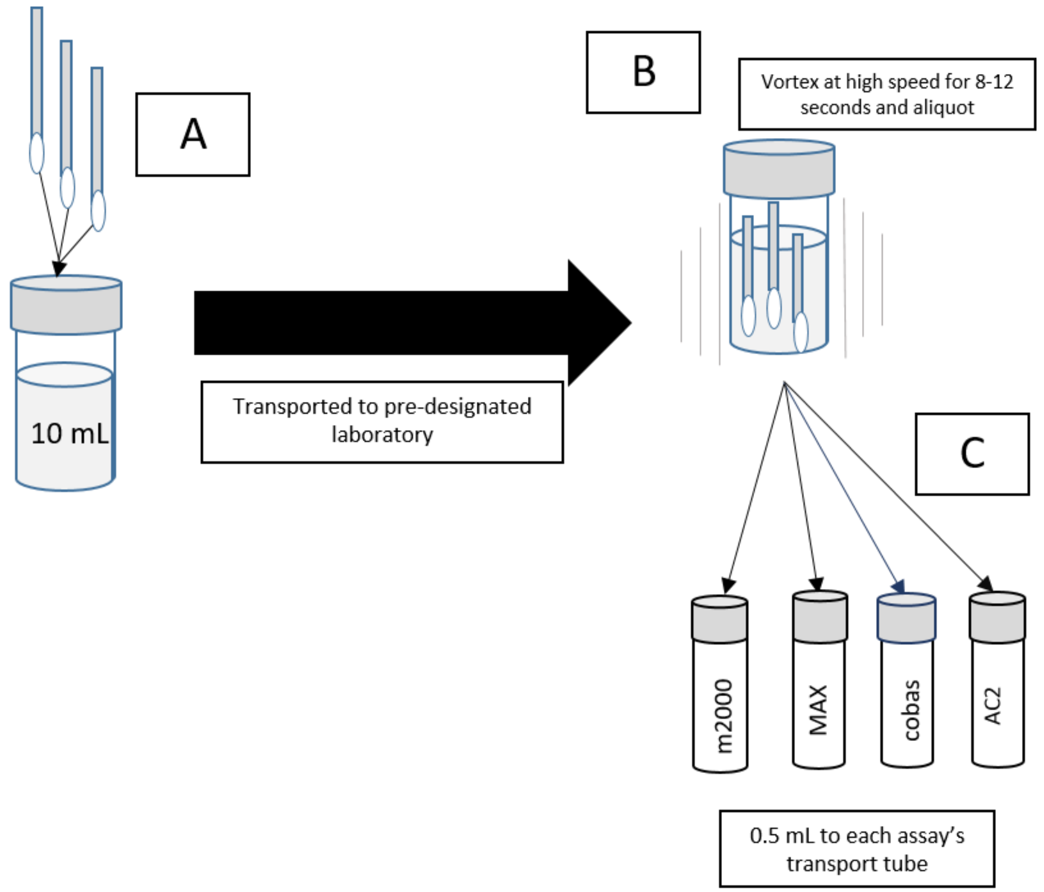

Figure legend: **[A]** 3 swabs per anatomical site will be collected at the clinic, and placed into 10 mL of PreservCyt. **[B]** In the laboratory the samples were vortexed at high speed for 8-12 seconds. 0.5ml aliquots will then be immediately placed into Abbott m2000 BD Max as well as the 4th NAAT transport tube. 0.75 mL was transported without buffer to labs performing Roche cobas testing where 0.5 mL was added to 1 mL of PreservCyt. **[C]** Abbott transport tubes contain 1.2 ml (final dilution1:2.4) MAX UVE contains 1.5 mL (final dilution 1:3); AC2 transport tubes contain 2.9 mL (final dilution 1:5.8); and cobas samples were added to 1.0 mL of PreservCyt since the assay runs directly from PreservCyt samples (final dilution 1:3).

Table S1. Distribution of Results by Number of Positive Assays by Organism and Sample Type

Organism/Sample Type	M2000	MAX	COBAS	AC2	N
CT Rectal	+	+	+	+	**115**
	+	+	+	-	**1**
	+	+	-	+	**3**
	+	-	+	+	**0**
	-	+	+	+	**16**
	+	+	-	-	**0**
	+	-	+	-	**0**
	-	+	+	-	**2**
	+	-	-	+	**1**
	-	+	-	+	**3**
	-	-	+	+	**6**
	+	-	-	-	**0**
	-	+	-	-	**6**
	-	-	+	-	**7**
	-	-	-	+	**16**
	-	-	-	-	**2058**
	NR*	-	-	-	**75**
	NR	NR	NR	NR	**23**
	-	NR	-	-	**16**
	NR	NR	-	-	**8**
	-	-	NR	-	**7**
	NR	-	NR	-	**7**
	-	NR	NR	NR	**5**
	NR	+	+	+	**4**
	-	INVALID**	-	-	**3**
	-	-	-	NR	**1**
	-	-	+	INVALID	**1**
	-	+	+	INVALID	**1**
	-	NR	+	-	**1**
	+	NR	NR	NR	**1**
	NR	-	-	+	**1**
	NR	-	+	+	**1**
	NR	NR	+	+	**1**
GC Rectal	+	+	+	+	**88**
	+	+	+	-	**0**
	+	+	-	+	**0**
	+	-	+	+	**1**

	-	+	+	+	**7**
	+	+	-	-	**0**
	+	-	+	-	**1**
	-	+	+	-	**0**
	+	-	-	+	**0**
	-	+	-	+	**1**
	-	-	+	+	**7**
	+	-	-	-	**1**
	-	+	-	-	**2**
	-	-	+	-	**6**
	-	-	-	+	**5**
	-	-	-	-	**2118**
	NR	-	-	-	**78**
	NR	NR	NR	NR	**23**
	-	NR	-	-	**16**
	NR	NR	-	-	**9**
	-	-	NR	-	**7**
	NR	-	NR	-	**7**
	-	NR	NR	NR	**6**
	-	INVALID	-	-	**2**
	NR	+	+	+	**2**
	+	INVALID	+	+	**1**
	+	NR	+	+	**1**
	NR	-	+	-	**1**
**CT Throat**	+	+	+	+	**23**
	+	+	+	-	**1**
	+	+	-	+	**0**
	+	-	+	+	**0**
	-	+	+	+	**3**
	+	+	-	-	**0**
	+	-	+	-	**0**
	-	+	+	-	**2**
	+	-	-	+	**0**
	-	+	-	+	**0**
	-	-	+	+	**0**
	+	-	-	-	**2**
	-	+	-	-	**2**
	-	-	+	-	**2**
	-	-	-	+	**29**
	-	-	-	-	**2179**

	NR	-	-	-	**79**
	-	NR	-	-	**17**
	NR	NR	-	-	**9**
	NR	NR	NR	NR	**8**
	NR	-	NR	-	**7**
	-	-	NR	-	**6**
	-	NR	NR	NR	**6**
	NR	-	-	+	**6**
	-	INVALID	-	-	**4**
	-	-	-	NR	**1**
	NR	+	+	+	**1**
	-	-	INVALID	-	**1**
	NR	+	-	-	**1**
	NR	+	+	INVALID	**1**
**GC Throat**	+	+	+	+	**71**
	+	+	+	-	**1**
	+	+	-	+	**0**
	+	-	+	+	**3**
	-	+	+	+	**17**
	+	+	-	-	**0**
	+	-	+	-	**1**
	-	+	+	-	**1**
	+	-	-	+	**0**
	-	+	-	+	**0**
	-	-	+	+	**8**
	+	-	-	-	**1**
	-	+	-	-	**20**
	-	-	+	-	**14**
	-	-	-	+	**7**
	-	-	-	-	**2096**
	NR	-	-	-	**84**
	-	NR	-	-	**16**
	NR	NR	-	-	**8**
	NR	NR	NR	NR	**8**
	NR	-	NR	-	**7**
	-	NR	NR	NR	**6**
	-	-	NR	-	**5**
	-	INVALID	-	-	**2**
	-	-	-	NR	**2**
	NR	+	+	+	**2**

	-	-	+	INVALID	**1**
	-	+	+	INVALID	**1**
	NR	NR	+	+	**1**
	+	INVALID	+	+	**1**
	+	NR	+	+	**1**
	-	-	INVALID	-	**1**
	NR	+	-	-	**1**
	-	INVALID	-	+	**1**
	-	+	-	INVALID	**1**
	-	+	NR	-	**1**

***NR** refers to No Result (usually as a result of exclusions or protocol deviations such as improper handling)

****INVALID** refers to those samples that had both an initial result and a repeat result of invalid or indeterminate

Table S2. Performance Estimates by Gender and Symptom Status for Abbott m2000, BD MAX and Roche cobas

m2000

Site/Organism	Gender	Symptoms	# Positive / N	Sensitivity (95% CI)	Specificity (95% CI)
Rectal CT	Female	No	59/1096	91.5% (81.7 - 96.3%)	99.9% (99.4 - 100%)
		Yes	1/12	100% (20.7 - 100%)	100% (74.1 - 100%)
		Total	60/1108	91.7% (81.9 - 96.4%)	99.9% (99.4 - 100)
	Male	No	74/1188	71.6% (60.5 - 80.6%)	100% (99.6 - 100%)
		Yes	12/68	83.3% (55.2 - 95.3%)	100% (93.1 - 100%)
		Total	86/1256	73.3% (63.1 - 81.5%)	100% (99.7 - 100%)
	Transgender	No	1/24	100% (20.7 - 100%)	100% (85.1 - 100%)
		Yes	0/2		100% (34.2 - 100%)
		Total	1/26	100% (20.7 - 100%)	100% (86.2 - 100%)
Rectal NG	Female	No	22/1096	86.4% (66.7 - 95.3%)	100% (99.6 - 100%)
		Yes	0/12		100% (75.8 - 100%)
		Total	22/1108	86.4% (66.7 - 95.3%)	100% (99.6 - 100%)

	Male	No	72/1188	83.3% (73.1 - 90.2%)	99.8% (99.3 - 100%)
		Yes	9/68	100% (70.1 - 100%)	100% (93.5 - 100%)
		Total	81/1256	85.2% (75.9 - 91.3%)	99.8% (99.3 - 100%)
	Transgender	No	2/24	100% (34.2 - 100%)	100.0% (84.5 - 100%)
		Yes	1/2	100% (20.7 - 100%)	100.0% (20.7 - 100%)
		Total	3/26	100% (43.9 - 100%)	100% (85.1 - 100%)
Oropharyngeal CT	Female	No	12/1091	91.7% (64.6 - 98.5%)	99.9% (99.5 - 100%)
		Yes	0/17		100% (78.5 - 100%)
		Total	12/1108	91.7% (64.6 - 98.5%)	99.9% (99.5 - 100%)
	Male	No	16/1174	75.0% (50.5 - 89.8%)	99.9% (99.5 - 100%)
		Yes	1/82	100% (20.7 - 100%)	100% (95.3 - 100%)
		Total	17/1256	76.5% (52.7 - 90.4%)	99.9% (99.5 - 100%)
	Transgender	No	0/25		100% (85.7 - 100%)
		Yes	0/1		100% (20.7 - 100%)
		Total	0/26		100% (86.2 - 100%)

Oropharyngeal NG	Female	No	20/1091	80.0% (58.4 - 91.9%)	100% (99.6 - 100%)
		Yes	2/17	50.0% (9.5 - 90.6%)	100% (75.8 - 100%)
		Total	22/1108	77.3% (56.6 - 89.9%)	100% (99.6 - 100%)
	Male	No	69/1174	71.0% (59.4 - 80.4%)	99.8% (99.3 - 100%)
		Yes	11/82	81.8% (52.3 - 94.9%)	100% (94.6 - 100%)
		Total	80/1256	72.5% (61.9 - 81.1%)	99.8% (99.3 - 100%)
	Transgender	No	2/25	100% (34.2 - 100%)	100% (85.1 - 100%)
		Yes	0/1		100% (20.7 - 100%)
		Total	2/26	100% (34.2 - 100%)	100% (85.7 - 100%)

MAX

Site/Organism	Gender	Symptoms	# Positive / N	Sensitivity (95% CI)	Specificity (95% CI)
Rectal CT	Female	No	60/1096	96.7% (88.6 - 99.1%)	99.4% (98.7 - 99.7%)
		Yes	1/12	100% (20.7 - 100%)	100% (74.1 - 100%)
		Total	61/1108	96.7% (88.8 - 99.1%)	99.4% (98.7 - 99.7%)

	Male	No	73/1188	91.8%<br>(83.2 - 96.2%)	99.5%<br>(98.9 - 99.8%)
		Yes	12/68	100%<br>(75.8 - 100%)	100%<br>(93.1 - 0.0%)
		Total	85/1256	92.9%<br>(85.4 - 96.7%)	99.6%<br>(99.0 - 99.8%)
	Transgender	No	1/24	1000%<br>(20.7 - 100%)	100%<br>(85.7 - 100%)
		Yes	0/2		100%<br>(34.2 - 100%)
		Total	1/26	100%<br>(20.7% - 100%)	100%<br>(86.7 - 100%)
Rectal NG	Female	No	23/1096	87.0%<br>(67.9 - 95.5%)	99.8%<br>(99.3 - 100%)
		Yes	0/12		100%<br>(75.8 - 100%)
		Total	23/1108	87.0%<br>(67.9 - 95.5%)	99.8%<br>(99.3 - 100%)
	Male	No	70/1188	91.4%<br>(82.5 - 96.0%)	99.9%<br>(99.5 - 100%)
		Yes	10/68	100%<br>(72.3 - 100%)	100%<br>(93.4 - 100%)
		Total	80/1256	92.5%<br>(84.6 - 96.5%)	99.9%<br>(99.5 - 100%)
	Transgender	No	2/24	100%<br>(34.2 - 100%)	100%<br>(85.1 - 100%)
		Yes	1/2	100%<br>(20.7 - 100%)	100%<br>(20.7 - 100%)
		Total	3/26	100%<br>(43.9 - 100%)	100%<br>(85.7 - 100%)

**Oropharyngeal CT**	**Female**	**No**	13/1091	100%<br>(77.2 - 100%)	99.7%<br>(99.2 - 99.9%)
		**Yes**	0/17		100%<br>(79.6 - 100%)
		**Total**	13/1108	100%<br>(77.2 - 100%)	99.7%<br>(99.2 - 99.9%)
	**Male**	**No**	14/1174	100%<br>(78.5 - 100%)	99.8%<br>(99.4 - 100%)
		**Yes**	1/82	100%<br>(20.7% - 100%)	100%<br>(95.3 - 100%)
		**Total**	15/1256	100%<br>(79.6 - 100%)	99.8%<br>(99.4 - 100%)
	**Transgender**	**No**	0/25		100%<br>(85.7 - 100%)
		**Yes**	0/1		100%<br>(20.7 - 100%)
		**Total**	0/26		100%<br>(86.2 - 100%)
**Oropharyngeal NG**	**Female**	**No**	21/1091	95.2%<br>(77.3 - 99.2%)	99.2%<br>(98.4 - 99.6%)
		**Yes**	2/17	50.0%<br>(9.5 - 90.6%)	100%<br>(77.2 - 100%)
		**Total**	23/1108	91.3%<br>(73.2 - 97.6%)	99.2%<br>(98.4 - 99.6%)
	**Male**	**No**	68/1174	86.8%<br>(76.7 - 92.9%)	98.7%<br>(97.8 - 99.2%)
		**Yes**	11/82	90.9%<br>(62.3 - 98.4%)	98.5%<br>(92.1 - 99.7%)
		**Total**	79/1256	87.3%<br>(78.2 - 93.0%)	98.7%<br>(97.8 - 99.2%)

		**No**	1/25	100%<br>(20.7 - 100%)	100%<br>(85.7 - 100%)
	**Transgender**	**Yes**	0/1		100.0%<br>(20.7 - 100%)
		**Total**	1/26	100%<br>(20.7 - 100%)	100%<br>(86.2 - 100%)

cobas

Site/Organism	Gender	Symptoms	# Positive / N	Sensitivity (95% CI)	Specificity (95% CI)
**Rectal CT**	**Female**	**No**	61/1096	95.1%<br>(86.5 - 98.3%)	99.8%<br>(99.3 - 100%)
		**Yes**	1/12	0.0%<br>(0.0 - 79.4%)	100%<br>(72.3 - 0.0%)
		**Total**	62/1108	93.6%<br>(84.6 - 97.5%)	99.8%<br>(99.3 - 100%)
	**Male**	**No**	68/1188	97.1%<br>(89.9% - 99.2%)	98.6%<br>(97.8 - 99.2%)
		**Yes**	12/68	91.7%<br>(64.6 - 98.5%)	100%<br>(93.4 - 100%)
		**Total**	80/1256	96.3%<br>(89.6 - 98.7%)	98.7%<br>(97.9 - 99.2%)
	**Transgender**	**No**	1/24	100%<br>(20.7 - 100%)	100%<br>(85.7 - 100%)
		**Yes**	0/2		100%<br>(34.2 - 100%)
		**Total**	1/26	100%<br>(20.7 - 100%)	100%<br>(86.7 - 100%)

**Rectal NG**	**Female**	**No**	20/1096	100% (83.9 - 100%)	99.6% (99.0 - 99.9%)
		**Yes**	0/12		100% (74.1 - 100%)
		**Total**	20/1108	100% (83.9 - 100%)	99.6% (99.0 - 99.9%)
	**Male**	**No**	68/1188	98.5% (92.1 - 99.7%)	99.0% (98.2 - 99.4%)
		**Yes**	10/68	100% (72.3 - 100%)	100% (93.6 - 100%)
		**Total**	78/1256	98.7% (93.1 - 99.8%)	99.1% (98.3 - 99.5%)
	**Transgender**	**No**	2/24	100% (34.2 - 100%)	100% (85.1 - 100%)
		**Yes**	1/2	100% (20.7 - 100%)	100% (20.7 - 100%)
		**Total**	3/26	100% (43.9 - 100%)	100% (85.7 - 100%)
**Oropharyngeal CT**	**Female**	**No**	13/1091	100% (77.2 - 100%)	100% (99.6 - 100%)
		**Yes**	0/17		100% (79.6 - 100%)
		**Total**	13/1108	100% (77.2 - 100%)	100% (99.6 - 100%)
	**Male**	**No**	14/1174	100% (78.5 - 100%)	99.7% (99.1 - 99.9%)
		**Yes**	1/82	100% (20.7 - 100%)	100% (95.3 - 100%)
		**Total**	15/1256	100. (79.6 - 100%)	99.7% (99.2 - 99.9%)

	**Transgender**	**No**	0/25		100%<br>(86.2 - 100%)
		**Yes**	0/1		100%<br>(20.7 - 100%)
		**Total**	0/26		100%<br>(86.7% - 100%)
**Oropharyngeal NG**	**Female**	**No**	20/1091	100%<br>(83.9 - 100%)	99.7%<br>(99.2 - 99.9%)
		**Yes**	1/17	100%<br>(20.7 - 100%)	92.9%<br>(68.5 - 98.7%)
		**Total**	21/1108	100%<br>(84.5 - 100%)	99.6%<br>(99.0 - 99.9%)
	**Male**	**No**	62/1174	100%<br>(94.2 - 100%)	98.2%<br>(97.2 - 98.8%)
		**Yes**	11/82	100%<br>(74.1 - 100%)	98.5%<br>(92.1 - 99.7%)
		**Total**	73/1256	100%<br>(95.0 - 100%)	98.2%<br>(97.3 - 98.8%)
	**Transgender**	**No**	2/25	100%<br>(34.2 - 100%)	100%<br>(85.7 - 100%)
		**Yes**	0/1		100%<br>(20.7 - 100%)
		**Total**	2/26	100%<br>(34.2 - 100%)	1000%<br>(86.2 - 100%)
